# Supplementary material for: Tissue-specific in vivo transformation of plasmid DNA in Neotropical tadpoles using electroporation
Source: PLoS One. 2023 Aug 17;18(8):e0289361. doi: 10.1371/journal.pone.0289361 (PMC10434853; doi:10.1371/journal.pone.0289361)
Supplement: S1 File — (PDF) [file pone.0289361.s001.pdf]

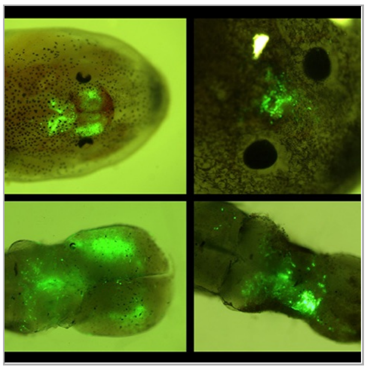

CUG4WTYW

Tissue-specific in vivo transformation of plasmid DNA in Neotropical tadpoles using electroporation V.(cug4wtyw)

Jesse Delia[1], Maiah Gaines-Richardson[1], Sarah C. Ludington[1], Najva Akbari[1], Cooper Vasek[2], Daniel Shaykevich[1], Lauren A O'Connell[1]

[1]Stanford University; [2]De Anza College

Lauren A O'Connell
Stanford University

Protocol Info: Jesse Delia, Maiah Gaines-Richardson, Sarah C. Ludington, Najva Akbari, Cooper Vasek, Daniel Shaykevich, Lauren A O'Connell . Tissue-specific in vivo transformation of plasmid DNA in Neotropical tadpoles using electroporation. **protocols.io** https://protocols.io/view/tissue-specific-in-vivo-transformation-of-plasmid-cug4wtywVersion created by Lauren A O'Connell

Created: May 19, 2023

Last Modified: May 20, 2023

PROTOCOL integer ID: 82172

Keywords: tadpole, brain, muscle, electroporation, plasmid

ABSTRACT

Electroporation is an increasingly common technique used for exogenous gene expression in live animals, but protocols are largely limited to traditional laboratory organisms. The goal of this protocol is to enable in vivo electroporation techniques in a diverse array of tadpole species. We explore electroporation efficiency in tissue-specific cells of five species from across three families of tropical frogs—poison frogs (Dendrobatidae), forest frogs (Aromobatidae), and glassfrogs (Centrolenidae). These species are well-known for their diverse social behaviors and intriguing physiologies that coordinate chemical defenses, aposematism, and/or transparency. Specifically, we examine the effects of electrical pulse and injection parameters on species- and tissue-specific transfection of plasmid DNA in tadpoles. After electroporation of a plasmid encoding green fluorescent protein (GFP), we found strong GFP fluorescence within brain and muscle cells that increases with the amount of DNA injected and electrical pulse number. We discuss species-related challenges, troubleshooting, and outline ideas for improvement. Extending in vivo electroporation to diverse amphibian species will offer a powerful approach to explore topics in genetics, behavior, and organismal biology.

ATTACHMENTS

Delia et al electroporation how to.mp4

GUIDELINES

These parameters are a suggested starting point. Optimizing the protocol for species-specific applications to maximize transfection efficiency is recommended.

MATERIALS

Ethyl 3-Aminobenzoate Methanesulfonate (Millipore Sigma Catalog #10521)
Sodium Bicarbonate (Millipore Sigma Catalog #S6014)
Josh's Frogs R/O Rx
Platinum Foil (Fisher Scientific Catalog #AA11509FF)
Lead Solder Wire (Amazon Catalog #B075WB98FJ)
Loctite Fun-Tak Mounting Putty Tabs (Amazon Catalog #1865809-12)
100 mm X 15 mm Petri Dishes (Fisher Scientific Catalog #FB0875713)
Serological Pipette (Fisher Scientific Catalog #12-567-600)
Electrical Tape (Fisher Scientific Catalog #19-047-280)
Micromanipulator (Sutter Catalog #MM-33)
Grass Instruments SD9 Square Pulse Stimulator
Disposable Paired 13 mm Subdermal Needle Electrodes (MFIMedical Catalog #RHL-RLSND121-1-0)
3.5" Replacement Glass Capillaries (Drummond Scientific Catalog #3-000-203-G/X)
Sutter Instrument Co P-97 (Sutter Catalog #P-P7)
Forceps (Fisher Scientific Catalog #12-000-157)
Mineral Oil (Millipore Sigma Catalog #M8410)
28 Gauge Metal Hub Blunt Point Needle (Fisher Scientific Catalog #14815616)
1 mL BD Disposable Syringe (Fisher Scientific Catalog #14-823-30)
Nanoject II Variable Volume Automatic Injector (Drummond Scientific Catalog #3-000-204)
pCMV-GFP (Addgene Catalog #11153)
Fast Green FCF (Millipore Sigma Catalog #F7258)
Kimwipes (Fisher Scientific Catalog #06-666)
Standard Disposable Transfer Pipettes (Fisher Scientific Catalog #13-711-7M)
Micro Detail Paint Brush
Stereomicroscope with a GFP filter

SAFETY WARNINGS

MS-222 is a respiratory irritant and the following personal protective equipment should be worn: labcoat, gloves and safety glasses.

BEFORE START INSTRUCTIONS

Consult with your local animal ethics board prior to experimentation.

Anesthesia Preparation

5m

1 Mix 0.02g ethyl 3-aminobenzoate methanesulfonate (MS-222) and 0.08g sodium bicarbonate with 60 mL tadpole water 5m

2 Store at 4C for up to one week

Electrode Set-Up for Targeting Muscle Fibers

25m

3 Remove the tips from two 5 mL serological pipettes using scissors 5m

4 Solder two ~ 5 mm X 8 mm pieces of platinum foil to separate electrical lead wires to make an electrode 5m

5 Run one electrode wire through each cut serological pipette and secure it with electrical tape 2m

6 Construct a platform out of clay evenly spread over the top of a Petri dish 2m

7 Embed the anode into the clay with the foil exposed near the center of the Petri dish 5m

8 Create a tadpole-sized (~ 1 cm) impression adjacent the anode foil 1m

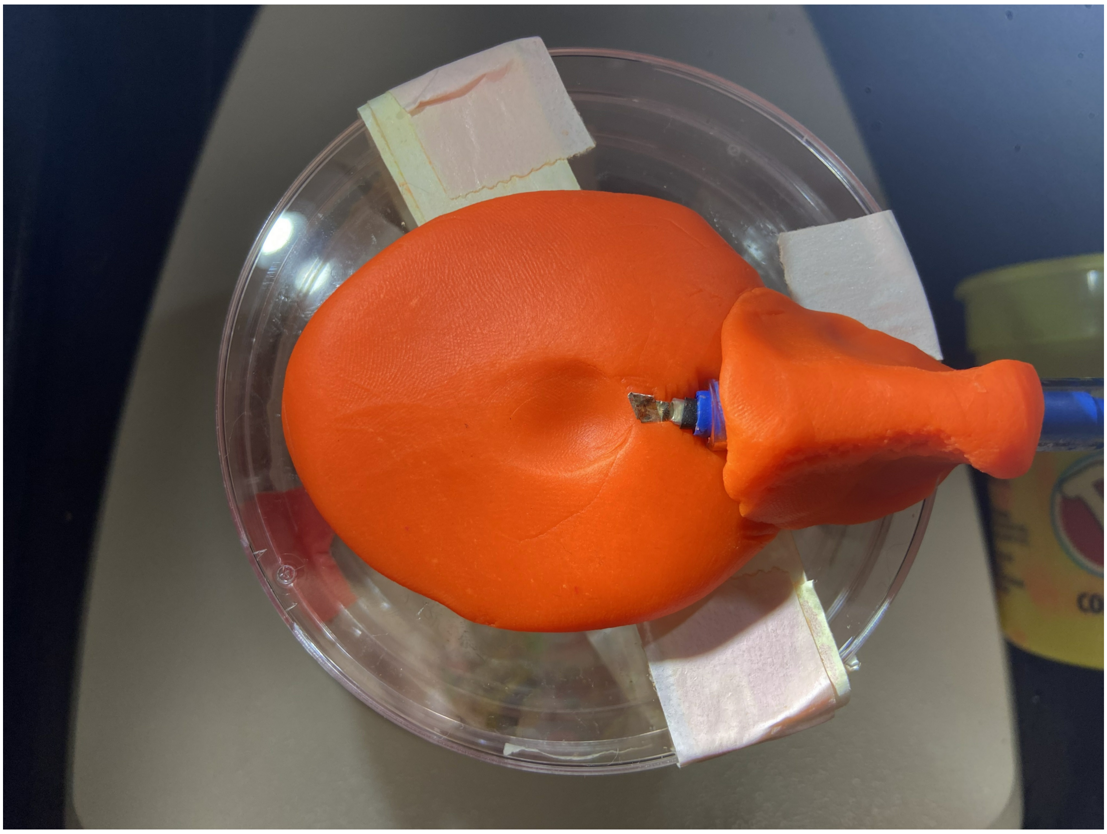

Figure 1. An anode embedded in a flat clay platform to hold tadpoles during the electroporation procedure.

9 Mount the cathode on a micromanipulator and bend the foil parallel to the anode 2m

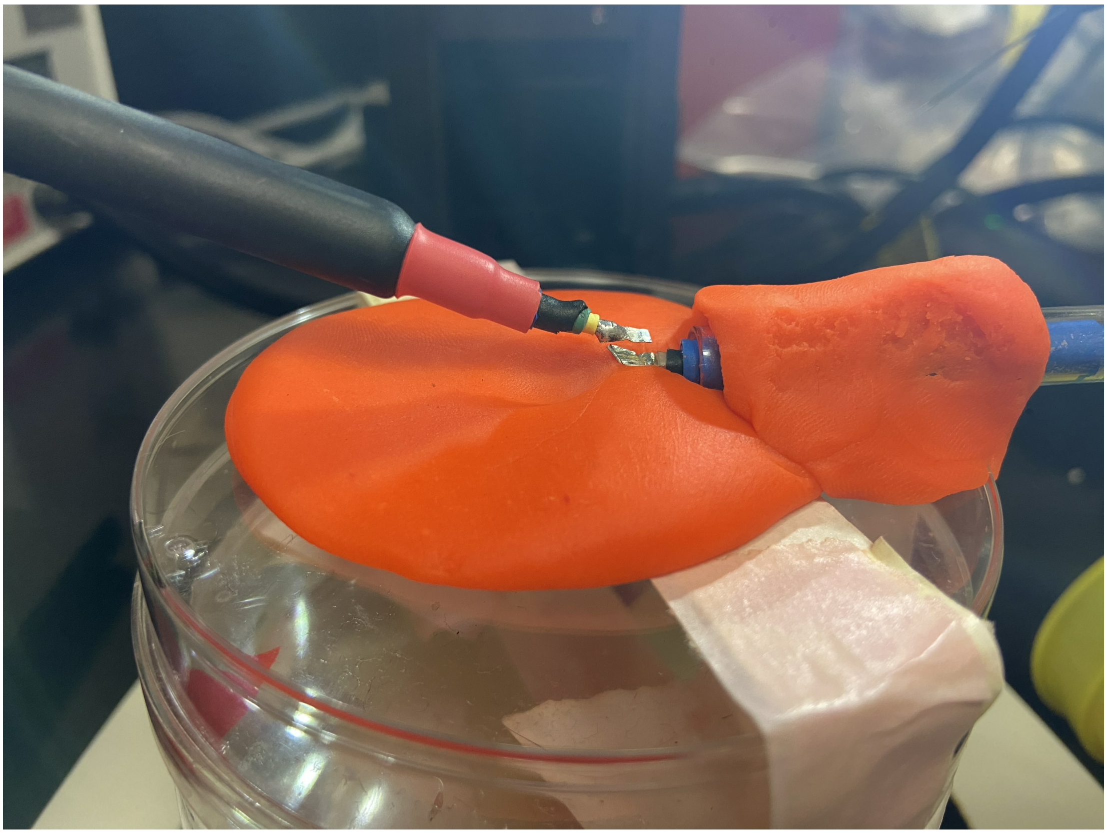

Figure 2. A cathode mounted on a micromanipulator in position to make contact directly above the anode during the electroporation procedure.

10 Set the stimulator parameters to square wave, 1 pps, 0.1 ms delay, 5 ms duration, and 30-50 V 1m

Electrode Set-Up For Targeting Brain Cells 25m

11 Construct a platform out of clay molded into the shape of a hill 5m

12 Fix the clay on the top of a Petri dish 1m

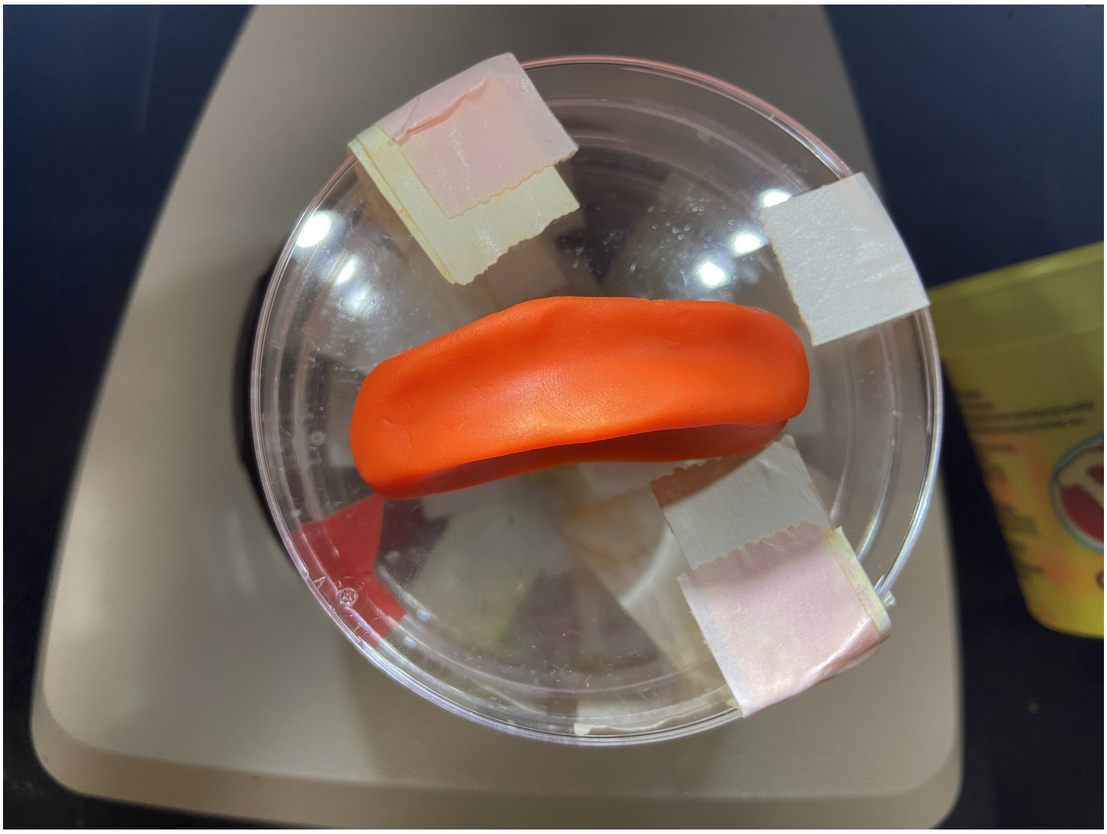

Figure 3. A clay platform molded into the shape of a hill to hold tadpoles during the electroporation procedure.

13 Remove the tips from two 1 mL serological pipettes using scissors 5m

14 Mount the serological pipettes side by side on the micromanipulator and fasten them together with electrical tape 5m

15 Run one needle electrode wire through each cut serological pipette 2m

16 Position the electrode needles ~ 1 mm apart and parallel to one another 5m

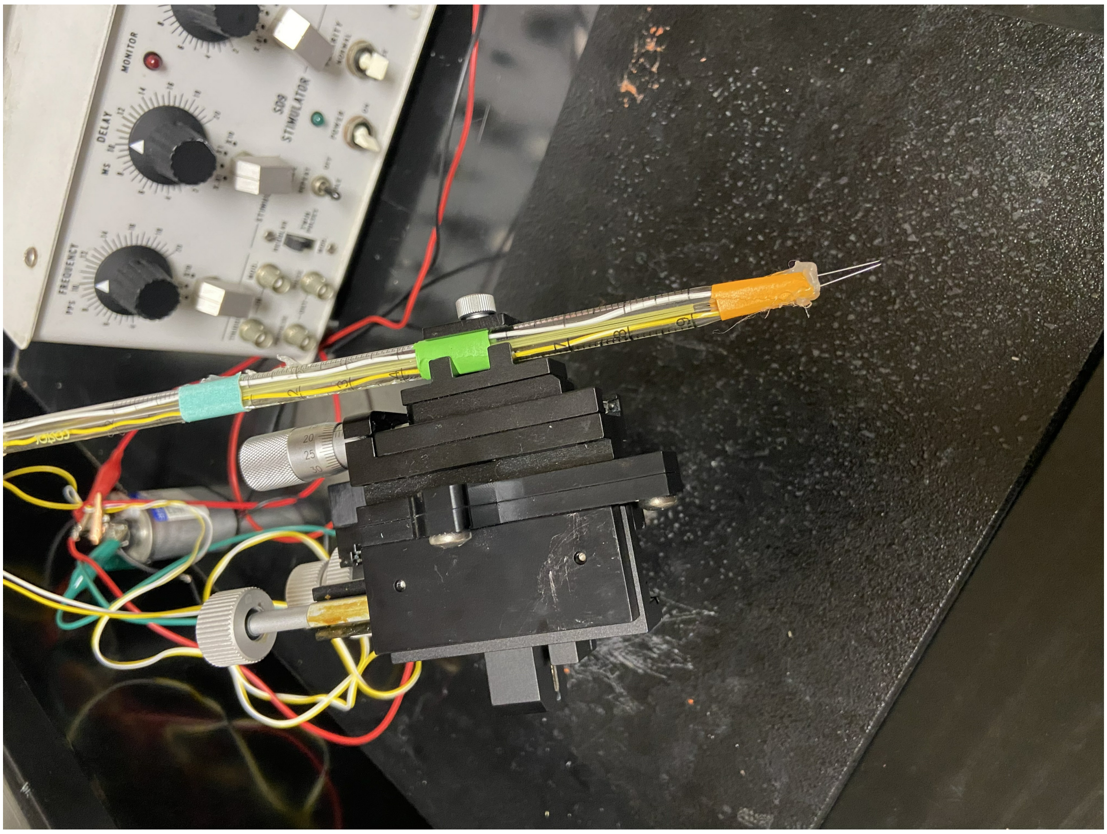

Figure 4. An assembled electrode mounted on a micromanipulator.

17 Set the stimulator parameters to 10 pps, 0.1 ms delay, 15 ms duration, and 30 V 1m

Note

These settings are a suggested starting point. Optimizing these parameters for each species, as the best pulse shape and settings may vary, is recommended.

Injection Set-Up

20m

18 Pull glass capillaries using a pipette puller 5m

19 Break the pipette tip at an angle using forceps to create a beveled tip 2m

20 Backfill the pipette with mineral oil using a 28-gauge needle and 1 mL syringe 2m

21 Place the pipette onto the injector plunger and tighten the collet 2m

22 Select an injection volume of 64.4 nl and set the injection rate to slow 2m

23 Empty enough mineral oil to load 2 uL of plasmid DNA solution 2m

24 Pipette 2 uL of plasmid DNA solution (0.25–0.27µg/µl) onto a piece of parafilm and mix with 0.2 2m

uL 0.01% Fast Green

25 Fill the pipette without introducing air bubbles 1m

26 Connect the electrode wires to the stimulator 1m

27 Place the platform under a dissection microscope with the electrode on one side and the injector on the other 1m

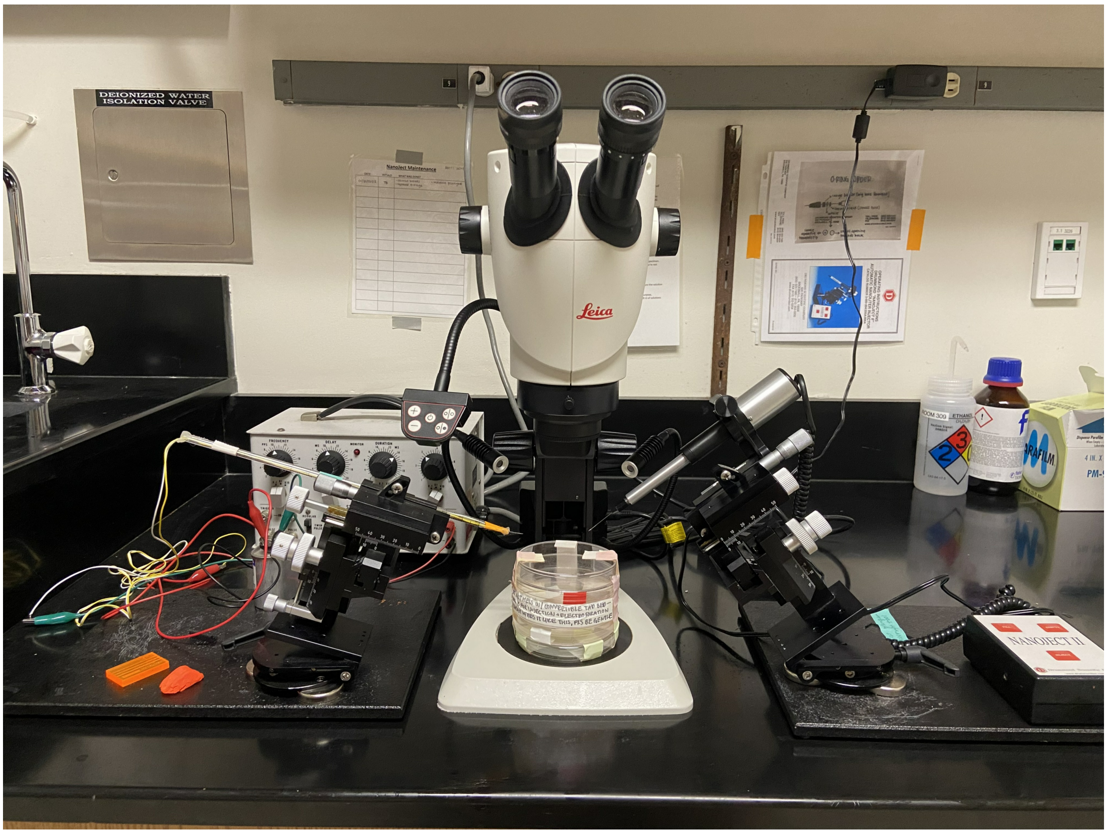

Figure 5. A dissection microscope focused on a tadpole injection platform that is placed between an electrode mounted on a micromanipulator and a microinjector mounted on a micromanipulator.

Electroporation 25m

28 Anesthetize the tadpole by placing it in a Petri dish of room temperature 0.03% MS-222 for 5 minutes 5m

29 Confirm the tadpole is fully sedated by checking for movement in response to stimuli 1m

30 Move the tadpole to the platform with a cut transfer pipette 2m

31 Adjust the position of the tadpole using a paintbrush 2m

31.1 For experiments targeting muscle fibers, place the tadpole flat on its side with its head in the depression and its tail on top of the anode

31.2 For experiments targeting brain cells, place the tadpole dorsal side up with its head in the depression

32 Orient the platform such that the head of the tadpole is facing toward the injector 1m

33 Lower the injector and insert the pipette into the target tissue 2m

33.1 For experiments targeting muscle fibers, insert the pipette into a tail myomere

33.2 For experiments targeting brain cells, insert the pipette into the brain ventricle

34 Inject the plasmid DNA with a 5-10 s interval between each injection 2m

34.1 For experiments targeting muscle fibers, deliver two injections

34.2 For experiments targeting brain cells, deliver three injections

35 Remove the pipette from the tadpole 1m

36 Orient the platform such that the head of the tadpole is facing toward the electrode 1m

37 Lower the electrode until it is in full contact with the target tissue 5m

37.1 For experiments targeting muscle fibers, the tail should lay on top of the anode and the cathode should press on the tail directly above the anode

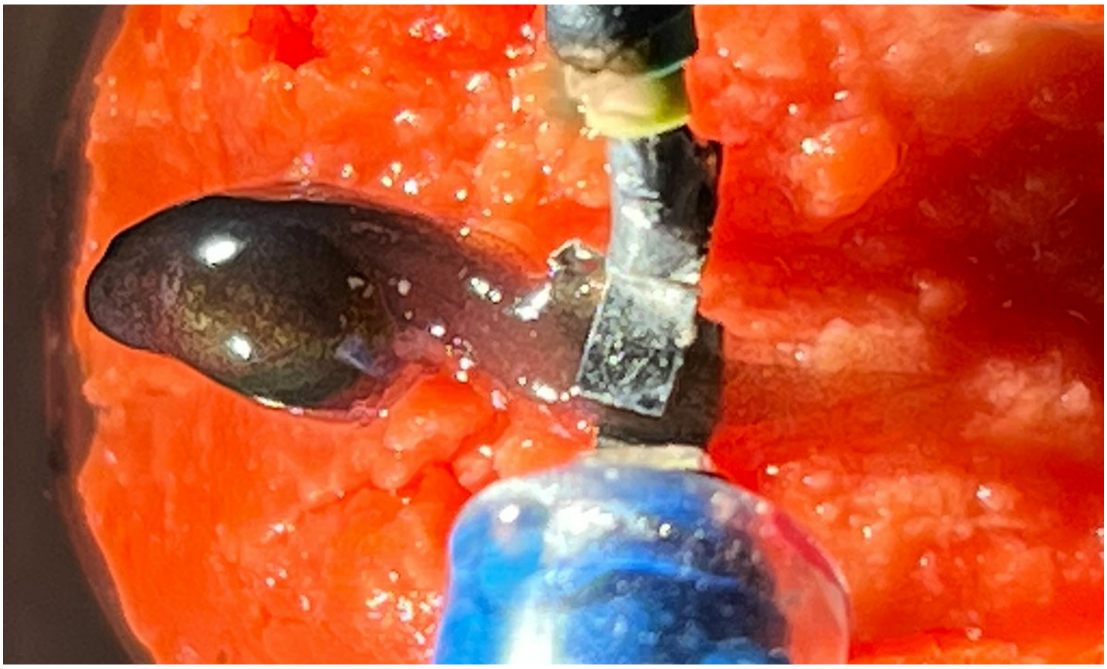

Figure 6. Muscle electroporation in the tail of a *Ranitomeya imitator* tadpole.

37.2 For experiments targeting brain cells, the electrode should be touching the head on either side of the brain

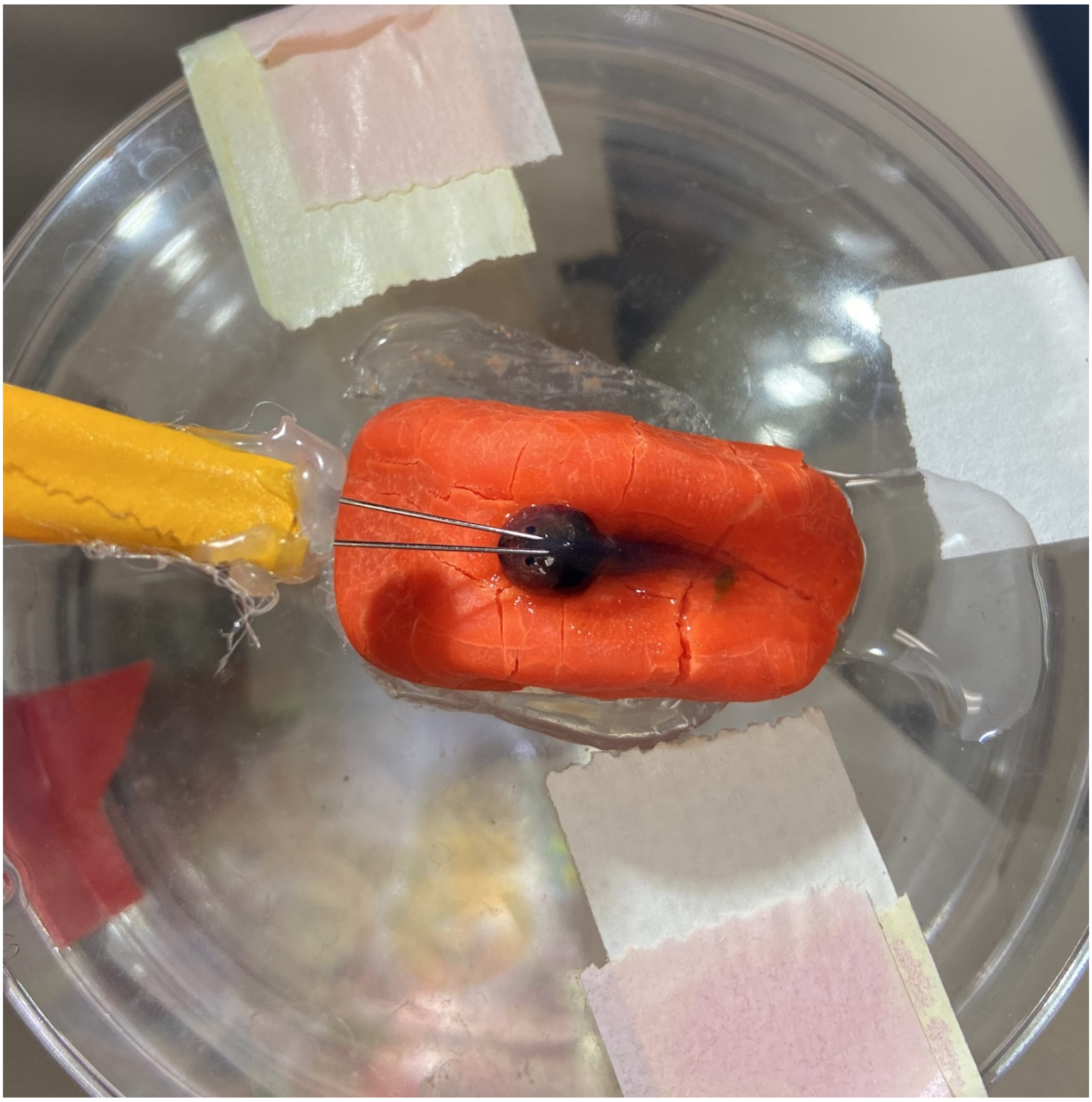

Figure 7. Brain electroporation in a *Ranitomeya imitator* tadpole with the electrode making contact with either side of the brain.

38 Deliver the electrical pulses 2m

> **Note**
>
> The pulse range for targeting brain cells is a suggested starting point. Optimizing the protocol for species-specific applications to maximize transfection efficiency is recommended.

38.1 For experiments targeting muscle fibers, deliver 4-8 double pulses with a 1 s interval between each set of pulses

38.2 For experiments targeting brain cells, deliver 4-10 pulses with a 1 s interval between each pulse

39 Transfer the tadpole to fresh tadpole water for several hours to recover 4h

40 Roughly 24 to 48 hours after electroporation, screen tadpoles for plasmid uptake by imaging GFP-positive cells

In Vivo Screening

20m

41 Anesthetize the tadpole by placing it in a Petri dish of room temperature 0.03% MS-222 for 5 minutes 5m

42 Move the tadpole to a new Petri dish with tadpole water and place under a stereomicroscope with a GFP filter 5m

43 Center the imaging field on the target tissue and capture the fluorescent image 10m

44 Transfer the tadpole to fresh tadpole water for several hours to recover 4h
