# Supplementary material for: Tissue-specific in vivo transformation of plasmid DNA in Neotropical tadpoles using electroporation
Source: PLoS One. 2023 Aug 17;18(8):e0289361. doi: 10.1371/journal.pone.0289361 (PMC10434853; doi:10.1371/journal.pone.0289361)
Supplement: Attachment — Submitted filename: Response to Review.docx [file pone.0289361.s004.docx]

**Response to Reviewer Comments (in bold).**

**We thank the reviewers for their comments and suggestions that have greatly improved this manuscript. We appreciate their time and commitment to improving our work. Thank you!**

**We respond to each comment below (in bold).**

*Reviewer #1*

*In their Lab Protocol entitled "Tissue-specific in vivo transformation of plasmid DNA in Neotropical tadpoles using electroporation," Jesse Delia and collaborators successfully adapted the electroporation technique to a range of non-model species tadpoles.
The electroporation technique has been used to transfer charged macromolecules, including fluorescent dyes and DNA, into living cells of Xenopus tadpoles for decades. In the present Protocol, the authors show that this technique is also suitable for transferring plasmid DNA into living cells of tadpoles of other species.
Although it is not very surprising that the electroporation technique works on tadpoles of different species, I believe that the present Protocol will be well received in the amphibian community.
The Protocol is well-written and straightforward. It will encourage researchers working with different amphibian species to use this powerful technique.

I would have loved it if you had also tried electroporating single cells. In your experiments, you perform bulk electroporations. Labeling specific cell types or cells of interest will be impossible with your method.
You could be more specific when stating what type of experiments can be done or improved using the described electroporation technique. Many experiments that I have in mind would necessitate a more precise and controlled electroporation of specific cells/ cell types.

I only have a few questions and suggestions that the authors could consider taking into consideration when revising their Protocol. I hope this helps to make some points more precise.*

**Response to general comments: We appreciate your kind and encouraging responses to our protocol manuscript, and for your helpful comments and time, which greatly improved our MS.**

**We agree that labeling specific cells would be beneficial for the community, but it is outside the scope of the current protocol. This protocol took many years to develop and applying your excellent idea would take us at least one year to implement with our current staffing and would need to be paired with a reliable brain atlas, which we are currently working on developing. We will continue to work in this direction for developing a protocol for single cell electroporation in the future. In the meantime, we hope more rapidly releasing this protocol will be useful for the amphibian community.**

*Specific points

You solely report the successful electroporation of plasmid DNA into cells. Have you tried to electroporate fluorescent dyes, calcium indicators, etc.?*

**Response: We have electroporated fluorescent calcium indicator dyes, but this is part of a larger project to be published separately, as it is not fully developed and requires different electroporation parameters that are not yet optimized. We have indicated that readers could apply this approach to a range of molecules in the discussion: “Further, this electroporation protocol can be further optimized by users to induce cellular uptake of various molecules useful for neuroscience research, as has been done in *Xenopus* tadpoles, including morpholinos, fluorescent dyes, and or single cell electroporation methods.”** *Some tadpole-specific electroporation Protocols are missing from your publication list.*

**Response: We have added more references of Xenopus electroporation to the introduction as follows: “Indeed, there are many protocols detailing methods optimized for Xenopus tadpoles that include electroporation of brains (Hewapathirane and Haas 2008; Haas et al. 2002; Bestman et al. 2006; Liu and Haas 2011; Sáenz de Miera et al. 2018; Schohl et al. 2020; Ruthazer et al. 2013), eyes (Ruthazer et al. 2013), and tails (Mochii et al. 2007; Mochii and Taniguchi 2009).”**

*While in Figure 2D, the position of the electrode on the tadpole is nicely visible, the other three subfigures of Figure 2 are not that helpful. The electrode position on the tadpoles is not clearly visible.
Is this a kinked tail of the tadpole sticking up in Figure 2B?*

**Response: We have adjusted the image in Figure 2B to fix the issue with the electrode, where the platinum sheet was previously sticking up. While we agree the photo in Figure 2D is very useful, we counter that showing the reader where to electroporate muscle (Figure 2B) as well as the general set-up (Figure 2A and C) is important for readers who are unfamiliar with this approach.** *Figure 3: When looking at the pictures you provide in Figure 3B, I cannot imagine how to count single cells. What are the borders of a single myocyte? How can you even be sure that these are all myocytes?*

**Response: In ours and other papers that targeted muscle cells in tadpoles, myocytes are confirmed and counted based solely on their morphology, size, and location within myomeres in tadpole tails (see ref 16, 24, etc). We did not perform traditional approaches to confirm the identity of myocytes (e.g., immunohistochemistry or electron microscopy). However, myocytes in tadpole myomeres are easily and commonly identified based on their distinct tubular morphology and large size (0.25 to over 1 mm in length; depending on animal size)—they are so large that they can sometimes be viewed in life without magnification (see new images in figure 3, and refs16,24,47,48). These cells are organized in parallel bundles that run the length of the muscle block within a myomere in the tail (see refs 16,24,47,48). The targeted tissue for electroporation—myomeres—are sequential blocks of muscle arranged in a zigzag pattern, running the length of the tadpole’s tail. To our knowledge, there are no other cells with this size or morphology that occur inside myomeres (e.g., those in epithelial tissues, fibroblasts, blood, nerves, etc.). However, to be transparent about the potential uncertainty here, we added new text (see bottom of this response).**

**Counting myocytes: We counted myocyte directly under a dissecting (fluorescent) microscope, where we could quickly adjust light levels, the animal’s position, and use forceps and probes to carefully examine the tail muscle to better delimit the edges of individual myocytes (but see disclaimer below). Please note that the images in figure 3 do not clearly show distinct cells due to brightness/exposure, which needs to be repeatedly adjusted based on individual cell’s position/depth and expression levels. We added new text for clarity and transparency on the limits of our method for cell counts. We also added new images to fig 3 to show the targeted region and myocyte morphology.**

**NEW TEXT on Lns 192-198:**

**We identified myocytes in *Xenopus* and the other species (below) based on their morphology using GFP-labeling and fluorescence microscopy. Myocytes are large cells that exhibit a distinct cylindrical morphology and are organized in parallel bundles within myomeres in tadpole tails [16,24,47,48]. We counted myocytes directly under a dissecting (fluorescent) microscope, where we could quickly adjust light levels, the animal’s position, and use forceps and probes to carefully examine the tail muscle to better delimit the edges of individual myocytes.**

**NEW TEXT on Lns 208-213:**

**Cell counts were likely influenced by species’ differences in the extent of skin pigments and muscle-tissue opacity, with the platanna and *Hyalinobatrachium* glassfrogs being highly transparent, and the poison frogs having more pigmented skin (see images in Figure 3). Importantly we conducted a within-species experimental design and combined mixed-modeling to compare within species’ effects. Our statistical model did not detect species-level or interaction effects (e.g., similar effect sizes across species, see results below).**

**NEW TEXT and images in fig 3 on Lns 314-327:**

**Note that specie’s differences in the extent of skin pigmentation and muscle-tissue opacity made counting cells difficult from images alone (i.e., adjusting brightness/exposure to capture specific cells can overexpose others and vice-versa depending on cell depth, position, and expression levels). Therefore, we counted myocytes directly under a dissecting (fluorescent) microscope, where we could quickly adjust light levels, the animal’s position, and use forceps and probes to carefully delimit the edges of individual myocytes at different depths. Cell counts were still limited to superficial cells in certain species due to pigmentation, and thus counts are not directly comparable across species—none the less, our experimental design did allow us to test treatment effects within species (and our GLMM modeling did not detect species-level effects). (C) An image showing the targeted region of muscle tissue in tadpole tails. Inset: myomeres are blocks of muscle tissue that are arranged in sequence across the tadpole’s tail; note the individual myocytes arranged in parallel within myomeres, which are large cylindrical cells that can be visible to the naked eye. (D) Brightfield (left) and fluorescent (right) images showing GFP-labeled myocytes within and delimited by the boarders of the myomeres.** *It is difficult to understand how you calculated the proportional area of transfected brain cells.*

**Response: This is a proportional calculation of brain area with GFP signal (gfp-positive area/total brain area). For clarity, we edited this text to (on Lns 254-256): “For analyses, we calculated the proportional area of brain tissue with GFP signal as the area with GFP signal divided by the total brain area (in pixels, in dorsal view).”**

*You state that non-model species of tadpoles can survive higher voltage, pulse number, and pulse duration if compared to Xenopus. Where do you show this?*

**Response: This text was based on our observations that xenopus experienced high levels of mortality in our experiments, whereas in the other species very few individuals died. As you point out, we did not include xenopus mortality in our analyses of non-model tadpole mortality. Therefore, we deleted the sentences from the manuscript. As we did analyze mortality for the non-Xenopus tadpoles, that text still remains in the manuscript.** *Line 260: …." using a using"….Please correct this part of the sentence.*

**Response:** **Corrected.** *Figure 4D: How can you be sure that the depicted cells are neurons? Where are these cells located in the brain?*

**Response: We have corrected this sentence (in fig 4 caption) to indicate brain cells rather than neurons. These cells are located in the hindbrain and this is now included in the manuscript. The text now reads as follows: “An image from two-photon microscopy showing individual GFP-labeled cells *in vivo* within the hindbrain of an *R. imitator* tadpole.”**

*Reviewer #2:*

*The manuscript by Delia et al is focus on plasmid DNA electroporation in non-model frog at tadpole stage. This work is really interesting because is one of the first description of this technique on non-model frog. The video and protocol are really well do and appreciable. This study can be accepted after major modification.*

**We thank you for your kind and helpful comments, encouraging review, and for your time.**

*Major comments
1- Xenopus is a classical frog model and it’s used as reference here to develop technics on non-model frog. It’s known that Xenopus Laevis embryos are commonly described as having a large size. Author can compare the size of non-model embryos at tadpole stage to Xenopus Laevis embryos?*

**Response: Many amphibians have big, beautiful eggs that develop in air. *Xenopus* eggs are small and aquatic. Comparing staging is difficult because *Xenopus* have a very detailed staging tables that’s specific to this species, whereas the non-model frogs have a crude simple table (Gosner 1960)—in which tadpoles are within a single stage for many months (theres a huge gap between hatching stage and then the development of limb buds at start of metamorphosis). Thus, comparing the size/stage of tadpoles between species is challenging.**

**However, we have added a few sentences on size and plasticity of tadpoles to the text (lines starting at 123):**

**“The species studied here lay larger (≥ 2 mm) but fewer eggs at oviposition compared to *Xenopus* (~1.2 mm). The non-model embryos also hatch larger and at more developed stages, although *X. laevis* can grow to a larger size at metamorphosis [37–41]. Many of these Neotropical species also exhibit considerable plasticity in hatching age, being capable of hatching at different development stages and hatching in response to environmental risk and parents [28,42–44].**

*2 – Authors used five various non-model frog in this study. Authors used some species for myogenic experiment and other for neural tissue. Authors could explain that ?*

**Response: We conducted these experiments during the first year of the Covid pandemic, during which there were numerous challenges to access to materials, personnel, and animals. The species sampling differences between experiments largely reflect differences in what species were available (breeding) at the time of each protocol development. We have added this text in the manuscript (starting at lines 128): “Differences in species sampling for each protocol were due to availability tadpoles during protocol development.”**

*3- In fig 3 authors described GFP expression in muscle cells. I’m not be able to see cells on picture and I’m not be able to localise fluorescent areas. Authors could add lower magnification to see where is located GFP on tadpole and higher magnification to see the counted cells.*

**Response: At your request, we have added the new images to figure 3.**

**Please note that we counted myocyte directly under a dissecting (fluorescent) microscope, where we could quickly adjust light levels, the animal’s position, and use forceps and probes to carefully examine the tail muscle to better delimit the edges of individual myocytes (but see disclaimer below). Please note that the images in figure 3 do not clearly show distinct cells due to brightness/exposure, which needs to be repeatedly adjusted based on individual cell’s position/depth and expression levels. We added new text for clarity and transparency on the limits of our method for cell counts. We also added new images to fig 3 to show the targeted region and myocyte morphology.**

*4- It is known that embryonic electroporation can be give mosaicism especially in nervous system but also in myogenic tissue. Author can discuss this point and precise when they talk about nervous system if GFP is often found in the same area or in various neural area.*

**Response:** **Thank you for this point. We have added the following sentence to our section on electrodes and tadpole shape: “Moreover, electroporation efficiency likely impacts the extent of mosaicism, as species with more globular shapes tend to have more sparse and asymmetric uptake in the brain.”** *Minor point
1- In phylogenetic tree author show one photo for two different species concerning Aromobatidae and Dendrobatidae. Authors can give a photo of each species.*

**Response: At your request, we have added new photos to figure 1. All studied species are now shown images in figure 1 save for R. variables, which is extremely similar to R. imitator—in fact the imitating poison frog (r imitator) mimics the color/pattern of R variables and they look identical to the untrained eye (these species are studied in the context of mimicry and speciation). We did not include R variables photos due to length of the figure.**

*2- Line 260: removed one “a using”.*

**Response:** **Corrected.**

*3- Some scale bars are missing for fig 3 and 4. Authors could add it ?*

**Response: Unfortunately, we don’t have accurate scale bars for those images due to an issue w the scope at the time this protocol was developed—during the pandemic when we couldn’t get it serviced.**
